# Overuse of plain abdominal radiography in emergency departments: a retrospective cohort study

**DOI:** 10.1186/s12913-019-3870-2

**Published:** 2019-01-14

**Authors:** Christophe L. Bertin, Simon Ponthus, Hari Vivekanantham, Pierre-Alexandre Poletti, Omar Kherad, Olivier T. Rutschmann

**Affiliations:** 1 Division of Emergency Medicine, Department of Community Medicine, Primary Care and Emergency Medicine, Geneva University Hospitals and School of Medicine, Geneva University, rue Gabrielle Perret-Gentil 2, 1205 Geneva, Switzerland; 2 Division of Internal Medicine, Hôpital de la Tour, Meyrin, Switzerland; 3 Division of Internal Medicine, Hôpital de la Tour and School of Medicine, Meyrin, Switzerland

**Keywords:** Plain X-ray, Abdomen, Emergency department

## Abstract

**Background:**

Plain abdominal radiography (PAR) is routinely performed in emergency departments (EDs). This study aimed to (1) identify the indications for PAR in EDs and compare them against international guidelines, (2) uncover predictors of non-compliance with guidelines, and (3) describe the use of additional radiological examinations in EDs.

**Methods:**

Retrospective cohort study in the EDs of two hospitals in Geneva, Switzerland, including all adult patients who underwent PAR in the EDs. Indications were considered “appropriate” if complying with guidelines. Predictors of non-compliance were identified by univariate and multivariate analyses.

**Results:**

Over 1 year, PAR was performed in 1997 patients (2.2% of all admissions). Their mean age was 59.7 years, with 53.1% of female patients. The most common indications were constipation (30.8%), suspected ileus (28.9%), and abdominal pain (15.3%). According to the French and American guidelines, only 11.8% of the PARs were indicated, while 46.2% of them complied with the Australian and British guidelines. On multivariate analysis, admission to the private hospital ED (odds ratio [OR] 3.88, 95% CI 1.78–8.45), female gender (OR 1.95, 95% CI 1.46–2.59), and an age >  65 years (OR 2.41, 95%CI 1.74–3.32) were associated with a higher risk of inappropriate PAR. Additional radiological examinations were performed in 73.7% of patients.

**Conclusions:**

Most indications for PAR did not comply with guidelines and elderly women appeared particularly at risk of being exposed to inappropriate examination. PAR did not prevent the need for additional examinations. Local guidelines should be developed, and initiatives should be implemented to reduce unnecessary PARs.

**Trial registration:**

ClinicalTrials.gov, identifier NCT02980081.

## Background

Plain abdominal radiography (PAR) is one of the most common radiological examinations performed in emergency departments (EDs), but its utility may be questioned for a number of reasons. Firstly, of the standard radiography techniques, it delivers one of the highest radiation doses (0.7 mSv as against 0.1 mSv for a chest radiography) [1]. Secondly, the information it provides is often insufficient to guide the clinician in management of the patient [2–7]. And lastly, new radiological techniques, such as low-dose computed tomography (CT), yield more useful data while exposing patients to similar radiation doses as PAR [6, 8–10].

In light of this, guidelines have been formulated to restrict the indications for PAR in EDs. The Australian Diagnostic Imaging Pathways (DIP) guidelines recommend that PAR be indicated only for suspected intestinal obstruction, perforation or foreign body and for following urinary stones [11]. The British Royal College of Radiologists (iRefer guidelines) restricts the indications for PAR to exacerbation of inflammatory bowel disease and screening for foreign bodies, obstruction, or perforation [12]. The utility of PAR in cases of suspected perforation is debatable, however, since the examination’s sensitivity is only 15% when analysed by non-radiologists [4]. The American College of Radiology (ACR) is more restrictive. Its guidelines consider that the examination is effective in cases of suspected foreign bodies and may be useful for following radiopaque urinary stone [13]. In all other indications, and particularly in cases of suspected obstruction, blunt abdominal trauma, and perforation, abdominal CT is deemed more appropriate than PAR. The French National Authority for Health (HAS) is also very restrictive, listing only two indications: ingested foreign body and suspicion of serious colitis in case of chronic inflammatory bowel disease [14].

These guidelines broadly belong to the “less is more” school of thought in medicine, which encourages clinicians and patients to recognise that too many diagnostic tests may lead to more risks than benefits. Like the “Choosing Wisely” project of the American Board of Internal Medicine, the Swiss Academy of Medical Sciences has asked medical societies to optimise resources by establishing lists of interventions that do not meet criteria for efficacy, appropriateness and cost-effectiveness. These interventions of limited utility should no longer be performed and may even no longer be reimbursed [15]. Given its diagnostic performance and poor risk–benefit ratio in most settings, PAR may fall under this category.

## Methods

This study’s main aim was to analyse indications for PAR in two EDs and to compare these indications against international guidelines.

### Study design and setting

This multicentric retrospective cohort study was conducted in two EDs in French-speaking Switzerland, Geneva University Hospitals (HUG) and Hôpital de La Tour (HDL), Geneva, Switzerland.

### Study population

Our study included all adult patients (> 16 years) who were prescribed PAR that was performed in the two EDs between 1 January and 31 December 2015. Any patient who was prescribed PAR that was not subsequently performed was excluded from our study.

### Study objectives

The primary objective was to determine the indications for PAR and compare them against the international guidelines. The secondary objectives were to identify any predictors for inappropriate use of PAR and to describe other imaging examinations performed in the ED (ultrasonography [US] or CT) as well as patients’ care pathways once they left the ED.

### Data collected

To achieve our main objective, indications for PAR were extracted from electronic patient medical records. The indications were classified as “appropriate” if they complied with international recommendations and as “inappropriate” in all other cases. Based on the British and Australian guidelines, the following indications were considered to be appropriate: suspected ileus, perforation, or foreign body; control following contrast medium injection; catheter verification; and follow-up of urinary stones [11, 12]. According to the French and American guidelines, the following indications were considered to be appropriate: suspected foreign body; control following contrast medium injection; catheter verification; and follow-up of urinary stones [13, 14]. This classification was arrived at by consensus agreement between two of the main investigators (CB, OR), and it was carried out on the basis of each international recommendation, from the most liberal to the most restrictive. To describe the study population and achieve our secondary objectives, the following data was collected from electronic clinical and administrative patient records: demographic characteristics (age, gender, date and time of admission), triage data (reason and acuity based on the Swiss Emergency Triage Scale [SETS]) [16, 17], care pathway (destination on discharge from the ED), primary diagnosis on discharge from the ED, and use of other radiological examinations during the stay in the ED. Lastly, in the HUG, the patients are treated in two different areas within the ED. The least acute emergencies are managed by a team of primary care physicians in the ambulatory section of the ED, whereas more acute emergency cases are managed by emergency physicians. This information was collected and included in our analyses.

### Statistics

Descriptive analyses (mean, standard deviation, median, interquartile range) were conducted to describe the study population. The characteristics of patients whose indication for PAR was appropriate according to the most restrictive guidelines (HAS and ACR) were compared against the characteristics of patients whose indication for PAR was inappropriate This comparison was performed by means of univariate and multivariate analyses using odds ratios (OR) and associated 95% confidence intervals (CI). The analyses were performed using the IBM SPSS statistics program for Windows, version 22 (IBM Corp., Armonk, New York, USA).

The study was approved by the research ethics committee of Geneva, Switzerland and registered on ClinicalTrials.gov (identifier NCT02980081).

## Results

### Study population

During 2015, 89′613 patients were seen in the two EDs. PAR (one view) was performed in 1997 patients (2.2%). The characteristics of patients who underwent PAR are described in Table 1. The study population comprised slightly more women than men (53.1% women vs. 46.9%), and mean age was similar in the two centres. Two peaks were noted in age distribution, one at 40 years and another at 90 years (Table 1).

**Table 1:** Characteristics of the patients admitted to the two emergency departments in Geneva and who underwent plain abdominal radiography

Characteristic	Total *N* = 1997
Age	
- mean (SD)	59.7 (22.2)
- median (IQR)	59 (41–80)
- > 65 years, *n* (%)	862 (43.1%)
- > 85 years, *n* (%)	314 (15.7%)
Male/female ratio (%)	46.9%/53.1%
Acuity level according to the SETS	
- 1	3.5%
- 2	29.5%
- 3	65.0%
- 4	2.0%
Reasons for consultation	
- Abdominal pain	45.2%
- Constipation	6.3%
- Vomiting/diarrhoea	5.6%
- Gastrointestinal bleeding	2.5%
- Foreign body ingestion	1.5%
- Renal compartment pain	8.4%
- Urological disorder	2.8%
- Acute confusion	1.5%
- Neurological disorder	3.4%
- Febrile state	2.1%
- Diminished general status	6.1%
- Cardiopulmonary disorder	6.9%
- Trauma	1.7%
- Others	6.0%

*SETS* Swiss Emergency Triage Scale

### Indications and compliance with international guidelines

The three most common indications observed were constipation, suspected ileus, and abdominal pain (Table 2). On the basis of the most restrictive French and American guidelines (HAS and ACR), 88.2% of indications were inappropriate. When the more liberal British and Australian recommendations were considered, 53.8% of indications were still non-compliant (Table 2).

**Table 2:** Indications for plain abdominal radiography

	Total *N* = 1997
Indications	
- Urinary stone	92 (4.6%)
- Suspected ileus	577 (28.9%)
- Abdominal pain	305 (15.3%)
- Constipation/coprostasis	617 (30.8%)
- Control after CM injection	28 (1.4%)
- Control catheter/probe	33 (1.6%)
- Suspected perforation	110 (5.5%)
- Follow-up	117 (5.9%)
- Foreign body	57 (2.9%)
- Non-specific transit disorders	38 (1.9%)
- Others	23 (1.2%)
Valid indications according to HAS	235 (11.8%)
Valid indications according to ACR	235 (11.8%)
Valid indications according to DIP	922 (46.2%)
Valid indications according to iRefer (RCR)	922 (46.2%)

*HAS* Haute Autorité de Santé – France, *ACR* American College of Radiology, *DIP* Diagnostic Imaging Pathway – Western Australia, *RCR* Royal College of Radiologists, *CM* contrast medium

On univariate analysis, admission to the private HDL, female gender, and an age over 65 years were associated with a higher risk of undergoing inappropriate PAR as defined by the restrictive guidelines of the HAS and ACR (Table 3). In the HUG, being treated in the ambulatory section of the ED was associated with a higher risk of inappropriate indications. These associations remained significant on multivariate analysis (Table 3).

**Table 3:** Predictors of inappropriateness of plain abdominal radiography according to the French National Authority for Health (HAS) and the American College of Radiology (ACR)

Factors	Unadjusted ORs (95%CI)	Adjusted OR (95% CI)
Centre		
- HUG	Ref	Ref
- HDL	3.71 (1.72–7.99)	3.88 (1.78–8.45)
Age		
- ≤ 65 years	Ref	Ref
- > 65 years	2.79 (1.97–3.69)	2.41 (1.74–3.32)
Gender		
- Men	Ref	Ref
- Women	2.10 (1.58–2.78)	1.95 (1.46–2.59)
Acuity level		
- 1	Ref	Ref
- 2	0.51 (0.18–1.46)	0.53 (0.18–1.53)
- 3	0.43 (0.16–1.20)	0.50 (0.18–1.42)
- 4	0.35 (0.09–13.2)	0.39 (0.10–1.54)
Time of the day		
- Daytime	Ref	Ref
- Nighttime	1.28 (0.93–1.77)	1.35 (0.97–1.89)
Weekend		
- Week	Ref	Ref
- Weekend	0.84 (0.62–1.13)	0.83 (0.61–1.13)
Ambulatory emergency		
- No	Ref	
- Yes	1.79 (1.19–2.69)	

*HUG* Geneva University Hospitals, *HDL* Hôpital de La Tour, *OR* odds ratio, *95% CI* 95% confidence interval, *Ref* reference

### Further radiological examinations, diagnosis and destination

An additional examination (US or abdominal CT) was performed in 73.7% of patients (Table 4). When we analysed the two most common reasons for prescribing PAR (suspected constipation and ileus), we noted that when PAR was performed because of suspected constipation (617 patients), the physicians ordered another imaging examination in 78.1% of cases, abdominal CT in 110 patients (17.8%), abdominal US in 468 patients (75.9%), and both in 96 patients (15.6%). These patients left the ED with a diagnosis of constipation in 30.5% of cases and non-specific abdominal pain in 24.1% of cases (Table 5). Of those who left with a diagnosis of constipation, an additional radiological examination had been performed in 80.3% of them. Similarly, when PAR was performed because of suspected ileus (577 patients), an additional examination was performed in 393 patients (68.1%), including 290 abdominal CT scans (50.3%). In the end, the diagnosis of ileus was made in only 79 of those patients (13.7%).

**Table 4:** Additional imaging examinations performed at the emergency department

	Total
Abdominal US	1091 (54.6%)
Abdominal CT scan	752 (37.7%)
Abdominal US or CT scan	1472 (73.7%)
Abdominal US and CT scan	371 (25.5%)

*US* ultrasonography, *CT* computed tomography

**Table 5:** Diagnoses and destination following discharge from emergency department

	Total
Non-specific abdominal pain	540 (27.0%)
Constipation	326 (16.3%)
Ileus	111 (5.6%)
Gastroenteritis	97 (4.9%)
Gastritis/ulcer	55 (2.7%)
Diverticulitis	26 (1.3%)
Abdominal foreign body	32 (1.6%)
Gastrointestinal bleeding	28 (1.4%)
Hepatic/biliary disorder	29 (1.5%)
Other abdominal disorder	49 (2.5%)
Urinary lithiasis	179 (9.0%)
Urinary infection	55 (2.8%)
Urological disorder	40 (2.0%)
Confusional state	45 (2.3%)
Respiratory disorder	94 (4.7%)
Rheumatological disorders	26 (1.3%)
Other extra-abdominal disorders	265 (13.3%)
Destinations	
- Home	747 (37.4%)
- Hospitalisation, surgery department	331 (16.6%)
- Hospitalisation, other department	919 (46.0%)

Non-specific abdominal pain (540 patients) and constipation (326 patients) accounted for 43.4% of all final diagnoses (Table 5). Of the patients whose final diagnosis was non-specific abdominal pain, only 19.1% underwent PAR alone.

## Discussion

This study primarily aimed to describe clinical practices among physicians working in the EDs of two hospitals in French-speaking Switzerland regarding the use of PAR. Over 1 year, PAR was performed in 2.2% of patients admitted to these departments. The three most commonly observed indications (75%) were abdominal pain, suspected ileus and constipation.

When compared against international guidelines, most of the indications for the PAR examinations performed in these departments were inappropriate, exposing the patients to unnecessary radiation. Only 46.2% of the indications complied with the Australian and British guidelines, and these are among the least restrictive [11, 12]. The French HAS and American ACR guidelines are more restrictive and were formulated by a group of health professionals that included radiologists and non-radiologists [13, 14]. According to these guidelines, more than 88% of the PAR examinations were performed for no recognised indication. The magnitude of this overuse may be surprising and possibly results from a lack of local guidelines and poor knowledge among physicians of the examination’s poor diagnostic performance and often downplayed potential risks. In addition, PAR is relatively cheap, delivers a lower radiation dose than standard CT, and has been used for many years as part of old decision algorithms. It has been shown that physicians sometimes find it hard to change their practices, especially when new rules require them to refrain from doing something [18].

Even though PAR offers no additional benefit than physical examination in non-trauma acute abdominal pain, this indication accounted for 15.3% of PAR examinations performed in our population. Recent guidelines on the management of acute abdominal pain do not include PAR anywhere in their management strategy [19], nor is PAR deemed useful in suspected appendicitis, biliary colic, or acute pancreatitis [20].

The indication for PAR in cases of suspected ileus continues to be the subject of debate. Only the French guidelines exclude this indication. The British, Australian, and American organisations consider the examination to be appropriate in this setting, mainly because of its good negative predictive value. In our two EDs, suspected ileus accounted for 28.9% of indications for PAR. In 31.9% of these patients, no other radiological examination was performed, which suggests that PAR made it possible to justifiably rule out the suspected diagnosis. Abdominal CT was the most frequently requested examination in the other cases, exposing the patient to two consecutive radiation sequences. Thus, it is reasonable to wonder whether this strategy is effective and whether the CT examination should not be performed first when ileus is suspected. To avoid excessive, dangerous radiation, low-dose CT might be an alternative for identifying not only the signs of obstruction but also its cause [21].

Suspected constipation accounted for 30.8% of indications for PAR in our population. This practice is not founded on any scientific evidence, since there exists no correlation between the presence of stools in the colon and a diagnosis of constipation [12]. The diagnosis of constipation should be made based on anamnesis comprising a description of the stools and discomfort affecting quality of life. In our cohort of patients, PAR was followed by another radiological examination (CT or US) in nearly 80% of cases, thereby apparently confirming the poor utility of PAR in such settings.

Even though PAR does no longer belong to the management of renal colic in emergency settings, this indication still accounted for nearly 5% of the PAR examinations performed in our two EDs [22]. Its diagnostic performance and similar radiation dose to PAR make low-dose CT an examination of choice [9]. That said, PAR remains an indication for following patients with radiopaque stones, a setting that accounted for 5.9% of all indications in our cohort.

The diagnostic performance of PAR is generally poorly defined in these various indications and does not prevent additional radiological examinations. More than 70% of the patients were subjected to further examinations, either CT or US. The rate of additional exams was higher at the university hospital than in the private hospital. This could be explained by differences in acuity levels (patients more severe in the university hospital) and by the higher proportion of very elderly patients. In addition, echography is more easily available at the university hospital.

Along with its inadequate diagnostic performance, PAR raises issues of quality of care, costs, and patient safety. The number of radiological examinations performed in EDs has exploded in recent years, exposing patients repeated radiation [23]. According to some specialists, between 1.5 and 2% of cancers diagnosed in the United States may be linked to excessive radiation exposure [24, 25]. Hence, emergency physicians have a duty to prevent unnecessary radiation. Lastly, given that each PAR examination has a mean cost of CHF 110, nearly CHF 200,000 could be saved each year if the most restrictive guidelines are applied.

Therefore, more reasonable and appropriate use of PAR in our EDs would perfectly accord with the principle of “less is more.” Cost reduction is not a priority in this approach, but it may have a positive spin-off effect when it coincides with the patient’s interests. Although PAR is not listed among the top five examinations identified by North American emergency medicine societies in the “Choosing Wisely” programs [26, 27], special efforts need to be made to restrict this examination in Switzerland.

Our study has several limitations. Because of the retrospective nature of our data collection, we were unable to closely analyse the emergency physicians’ clinical rationale. Nor was it possible to identify the indications in more detail beyond what the clinician noted on the radiology prescription slip. In addition, the lack of a real gold standard and the disparity among international guidelines represent an issue for determining the appropriateness of PAR. Besides, the conclusions of the PAR reports were not sufficiently standardized to assess the diagnostic performance of PAR and to compute it’s sensitivity and specificity. Furthermore, the study was conducted in two centres only, a factor that may limit the generalisability of our results. However, given the lack of national guidelines and considerable differences in practice between the private hospital and the public teaching hospital department, we may reasonably suppose that similar variability may be found in other hospitals. Lastly, despite detailed analysis of the records, the information we obtained was insufficient to clearly establish how decisive PAR had been in decision-making or diagnosis. Only a prospective study would enable such analysis. We were likewise unable to identify any characteristics of prescribers that might be predictive of poor use of the examination.

## Conclusions

Despite international guidelines, PAR is too frequently performed in our EDs. Establishing local guidelines and training programs for emergency physicians or general practitioners who work in these departments is crucial for reducing the number of unnecessarily prescribed examinations.

## Abbreviations

ACR: American college of radiology
CHF: Swiss francs
CI: Confidence interval
CT: Computed tomography
DIP: Diagnostic imaging pathway
ED: Emergency department
HAS: Haute Autorité de Santé (National Authority for Health, France)
HDL: Hôpital de la Tour
HUG: Hôpitaux Universitaires de Genève (Geneva University Hospitals)
OR: Odds ratio
PAR: Plain abdominal X-ray
US: Ultrasonography
